# Carcinogenic Activity and Risk Assessment of PAHs in Ambient Air: PM_10_ Particle Fraction and Bulk Deposition

**DOI:** 10.3390/toxics11030228

**Published:** 2023-02-27

**Authors:** Ivana Jakovljević, Iva Smoljo, Zdravka Sever Štrukil, Gordana Pehnec

**Affiliations:** Environmental Hygiene Unit, Institute for Medical Research and Occupational Health, 10000 Zagreb, Croatia

**Keywords:** toxic PAHs, cancer risk assessment, ecological risk assessment, bulk deposition, particulate matter

## Abstract

This paper present seasonal variation in the equivalent concentration (BaP_eq_) of PAHs in order to assess the potential cancer risk for two different groups of residents via ingestion, dermal contact and inhalation pathways. The possible ecological risk caused by PAH atmospheric deposition based on risk quotient was also estimated. A bulk (total, wet and dry) deposition and PM_10_ particle fraction (particles with an equivalent aerodynamic diameter < 10 µm) were collected from June 2020 to May 2021 at an urban residential location in the northern part of Zagreb, Croatia. The monthly average of total equivalent BaP_eq_ mass concentrations of PM_10_ varied from 0.057 ng m^−3^ in July to 3.656 ng m^−3^ in December; the annul ∑BaP_eq_ average was 1.348 ng m^−3^. In bulk deposition, ∑BaP_eq_ mass concentrations varied from 1.94 to 57.60 ng L^−1^. In both investigated media, BaP had the highest contribution in carcinogenic activity. For PM_10_ media, dermal absorption implied the greatest potential cancer risk, followed by ingestion and inhalation. For bulk media, a moderate ecological risk for BaA, BbF and BaP was observed according to the risk quotient approach.

## 1. Introduction

In the atmosphere, particulate matter (PM) can originate from natural sources (forest fires, volcanic eruptions, etc.), but they also may be a consequence of human activity (industry, vehicle, construction, etc.) [1]. Airborne particles can adsorb more or less toxic components, such as polycyclic aromatic hydrocarbons (PAHs), heavy metals, polychlorinated biphenyls (PCB), etc. Activities such as combustion of carbon-based fuels, fossil fuels, wood, peat, biomass and waste can cause PAH emission into the atmosphere [2]. PAHs can have a harmful impact on human health and ecosystems due to their ubiquitous occurrence and mutagenic and carcinogenic characteristics. The International Agency for Research on Cancer (IARC) and US Environmental Protection Agency (US EPA) classified PAHs into three groups, Group 1, 2A and 2B, according to their influence on humans [3]. The most investigated PAH, benzo(a)pyrene (BaP), is classified as carcinogenic to humans (Group 1). Dibenzo(ah)anthracene (DahA) is classified into group 2A—probably carcinogenic to humans; possibly carcinogenic to humans (2B) are benzo(a)anthracene (BaA), benzo(b)fluoranthene (BbF), benzo(k)fluoranthene (BkF), benzo(j)fluoranthene (BjF), chrysene (Chry) and indeno(1,2,3,-cd)pyrene (IP), while benzo(ghi)peylene (BghiP), fluoranthene (Flu) and pyrene (Pyr) are not classified [3,4]. The carcinogenic consequences of PAHs are due to their capability to bind to DNA [2,5,6]. Previous studies have shown a positive correlation between levels of PAH-DNA adduct formation in different tissues and PAH doses [7,8]. Humans can be exposed to PAHs through several routes: water, food, tobacco smoke, ambient air, pharmaceutical products, etc. Ambient air is one of the major sources of PAH intake [9]. Previous studies have shown that workers in coke production and coal gasification have an increased possibility of developing cancer during their lifetime [10,11]. A study by Boffetta et al. (1997) showed that chemistry sweeps and workers who used tar were dermally exposed to large amounts of PAHs [12]. Some laboratory studies on animals, where animals were exposed to definite concentrations of some PAHs over long periods, have shown that exposure to certain doses of PAHs could cause lung cancer from inhalation, stomach cancer from ingestion via food and skin cancer from dermal contact [13,14,15].

Atmospheric deposition is one of the main removal mechanisms of toxic compounds from the atmosphere to aquatic and terrestrial systems [16,17,18]. Atmospheric deposition occurs as dry, wet and gas deposition. Wet deposition refers to the process of deposition of pollutants from the atmosphere through precipitation or snow, while dry deposition involves the gravitational deposition of atmospheric particles. The bulk deposition is an operational definition, described as the total deposition of material (wet and dry) to a continuously open collector [19,20]. Over the past several decades, interest for organic composition of atmospheric deposition has increased [18,21,22,23,24,25,26,27,28,29,30,31,32]. PAHs in atmospheric deposition can affect human health directly by ingestion and dermal contact only in the areas where collected rainwater is used directly for washing or drinking. However, by dry and wet deposition PAHs reach waters, vegetation and soil and can have adverse effect on the environment, as well as enter the food chain. Therefore, the determination of PAHs in atmospheric deposition is important for assessing ecological risks. Unfortunately, measurements of PAHs in atmospheric deposition are much less frequent compared to PM.

Previous studies in Croatia were based mostly on the determination of PAH levels in particulate matter [33,34,35,36,37], and only a few studies have considered PAHs in deposition samples [17,18]. The carcinogenic activity of PAHs was estimated only for particulate matter at different locations in Croatia [36,38]. However, the corresponding health risk was estimated only for certain crossroad locations [33] and for the PM_1_ particle fraction (PM with equivalent aerodynamic diameter < 1 µm) [39]. Considering the ecological risk from PAHs, studies in the field are focused mainly on rivers, sediments and soil [40,41,42,43] or, rarely, rainwater [44]. To the best of our knowledge, there is no study that assesses the ecological risk of toxic PAHs present in bulk deposition samples.

In this study, the main objectives were to investigate the temporal variation in PAHs’ carcinogenic activity by calculating the carcinogenic equivalent concentration (BaP_eq_) in the PM_10_ fraction of particulate matter (particles with an equivalent aerodynamic diameter < 10 µm) and bulk deposition. The PM_10_ fraction was chosen because Directive 2004/107/EC of the European Union, as well as several national and air quality standards, are principally focused exclusively on PAHs bound to this fraction, so the data obtained in this study will be easy comparable with other similar studies and already published data [45,46]. The potential cancer risk for two different groups of residents via ingestion, dermal contact and inhalation pathways was assessed using data on PM_10_ exposure. Furthermore, this is the first investigation of possible ecological risk caused by PAHs in bulk deposition based on risk quotient calculations.

## 2. Materials and Methods

### 2.1. Sampling

In this study, samples of PM_10_ particle fraction and bulk deposition (wet and dry deposition were collected together) were collected from June 2020 to May 2021 at an urban residential site in the northern part of Zagreb, Croatia’s capital. The geographical coordinate of the measuring site was 45°50′9″ N, 15°59′4″ E. This area is characterized by low resident density and modest traffic. A previous study in this area showed that households mostly used gas for residential heating, but oil and wood were also in use for heating or cooking [36,37].

For sampling of the PM_10_ particle fraction on quartz filters (Whatman, QMA, 47 mm), low-volume sequential automatic samplers (SEQ 47/50, Sven Leckel) were used according to the EN 12, 341 standard (2.3 m^3^ h^−1^). Over a one-year period, 24-hour samples were collected and wrapped in aluminum foil and kept frozen until analysis [37,47]. During the same period, monthly samples (30 ± 2 days) of bulk deposition were collected using a bulk funnel-bottle collector constantly open to wet and dry deposition. The sampling procedure was described in the study by Šimić et al. (2020); briefly, an open 2.5 L cylindrical dark glass bottle was wrapped in aluminum foil, connected to a funnel with a diameter of 150 mm and set on a steel chassis 2 m above ground [17]. At the end of the sampling period and before samples storage, the funnel was cleaned with glass wool and rinsed with acetone. Bulk deposition samples were stored in darkness at 4 °C until analysis.

### 2.2. Analysis of PAHs in PM_10_ Particle Fraction

The PAH extraction procedure from PM_10_ particles was already described in previous papers [36,37,47]. Briefly explained, 24-hour filters were extracted with a solvent mixture of cyclohexane and toluene in an ultrasonic bath, separated from undissolved parts by centrifugation and evaporated to dryness in a mild stream of nitrogen. After evaporation, samples were re-dissolved in acetonitrile. Mass concentrations of PAHs in PM_10_ particle fraction were determined by an Agilent Infinity 1260 high performance liquid chromatograph coupled with a fluorescence detector, with acetonitrile as the mobile phase and a flow rate of 1 mL min^−1^. The following PAHs were determined: fluoranthene (Flu), pyrene (Pyr), benzo(a)anthracene (BaA), chrysene (Chry), benzo(b)fluoranthene (BbF), benzo(k)fluoranthene (BkF), benzo(a)pyrene (BaP), dibenzo(a,h)anthracene (DahA), benzo(ghi)perylene (BghiP) and indeno(1,2,3-cd)pyrene (IP). Five-point calibration curves were used ranging from 0.005 ng μL^−1^ to 0.08 ng μL^−1^ for Pyr, BaA, Chry, BkF, BaP and IP and from 0.01 ng μL^−1^ to 0.16 ng μL^−1^ for Flu, BbF, DahA and BghiP. The accuracy of the method was confirmed by analysis of urban dust 1649 b (NIST certificated reference material). The method accuracy ranged from 88% for Flu to 109% for BkF. The limit of detection (LOD) was from 0.001 ng m^−3^ for BaA to 0.03 ng m^−3^ for BjF.

The measurements of PAHs in the PM_10_ particle fraction were funded by the City of Zagreb, City Office for Economy, Energetics and Environment Protection, and are available to public at the Zagreb air quality monitoring network website (http://www1.zagreb.hr/kvzraka/index.htm accessed on 28 December 2022).

### 2.3. Analysis of PAHs in Bulk Deposition Samples

The detailed procedure for extraction and analysis of PAHs in bulk deposition samples was described in an earlier paper [17,18]. In brief, deposition samples were filtered using glass microfiber filters and extracted with n-hexane for 15 min in an ultrasonic bath. Filtrates were passed through SPE cartridges and eluted with n-hexane and dichloromethane. After dehydration of the eluate with anhydrous sodium sulphate, the eluate was reduced nearly to dryness under a nitrogen flow by a rotary evaporator. The reduced samples were then re-dissolved in 1 mL n-hexane, and 100 µL of the internal standard (IS) was added. The same PAH compounds as in the PM_10_ particle fraction were analyzed. Analysis was carried out by a gas chromatograph (Agilent Technologies 7890B, Santa Clara, CA, USA) coupled with a tandem mass spectrometer (Agilent Technologies 7000C, Santa Clara, CA, USA). The conditions of gas chromatograph and mass spectrometer were described in a previous paper [17,18]. Quantification limits (QL) for PAHs in bulk deposition were 0.080–0.298 ng mL^−1^.

### 2.4. Statistical Analysis

The statistical analysis was processed by Microsoft Excel and Statistica 13 (Tibco Software Inc., Palo Alto, CA, USA). Statistical significance was set at 5% (*p* < 0.05). Seasonal variances between warm and cold period for each PAH in the PM_10_ particle fraction and in bulk deposition were tested by Kruskal–Wallis analysis of variance test. Cancer risk and exposure dose for PAHs in PM_10_ particle was estimated using a model developed by the US EPA [48], while for evaluation of the ecosystem risk by toxic PAHs in bulk deposition, the risk quotient (RQ) was used.

### 2.5. Cancer Risk Assessment of PAHs

The cancer risk assessment was estimated as the possibility of developing all types of cancer from lifetime exposure to carcinogen compounds. The exposure dose (D) was estimated using a model developed by the US EPA [48]. As routes for human exposure to carcinogenic compounds from particulate matter can be through inhalation (D_inh_), dermal absorption (D_der_) and ingestion (D_ing_). Total cancer risk (Risk_tot_) was calculated by summing the risk from each exposure pathway (ingestion + dermal + inhalation). The health risk assessment performed in the present study was based on the guidelines defined by the United States Environmental Protection Agency (US EPA). The biometric and exposure parameters of the residence were assumed to be similar to those of the U. S. residents, so the values of parameters used in the calculation of risk assessment in this study were taken from the standard default exposure factors of the US EPA [49] and were used worldwide for calculations of risk assessment [1,50,51,52,53,54,55].

Risks were estimated for two different types of residents, children under the age of 6 and adults from 20 to 40 based on EPA guidance on selecting age groups for monitoring and assessing exposures to environmental contaminants [56]. For particulate matter the dose received through these three pathways was calculated by the following equations:

(1) $$D_{\mathrm{ing}}=\frac{C\;\times \;\mathrm{CF}\;\times \;\mathrm{IRs}\;\times \;\mathrm{EF}\;\times \;\mathrm{ED}}{\mathrm{BW}*\mathrm{AT}}$$

(2) $$D_{\mathrm{der}}=\frac{C\;\times \;\mathrm{CF}\;\times \;\mathrm{SA}\;\times \;\mathrm{AF}\;\times \;\mathrm{ABS}\;\times \;\mathrm{EF}\;\times \;\mathrm{ED}}{\mathrm{BW}*\mathrm{AT}}$$

(3) $$D_{\mathrm{inh}}=\frac{C\;\times \;\mathrm{IR}\alpha \;\times \;\mathrm{ET}\;\times \;\mathrm{EF}\;\times \;\mathrm{ED}}{\mathrm{PEF}*\mathrm{BW}*\mathrm{AT}}$$

where C is the concentration of the toxic compound (mg kg^−^^1^) estimated on the basis of toxic equivalency factors (TEFs) from the literature. Many TEF schemes are established by different authors [57,58,59]. The toxic equivalent factors proposed by Nisbet et LaGoy (1992) [58] are often used and were applied in earlier studies in Croatia [36,38]. For calculating the concentration of toxic compound (C), the carcinogenic activities of individual PAHs in the air are expressed relative to the potency of BaP. Concentrations of toxic compounds were calculated by multiplying the mass concentration of an individual PAH with its own toxic equivalency factor. CF is the conversion factor (10^−^^6^ kg mg^−^^1^) [48]; IRs is the ingestion rate (mg day^−^^1^); EF is the exposure frequency (day year^−^^1^); ED is the exposure duration (year), BW body weight (kg); AT is average time (day); SA is exposed skin area (cm^2^); AF is skin adherence factor (mg cm^−^^2^); ABS is absorption factor (unitless); IRα is inhalation rate (m^3^ h^−^^1^); ET is exposure time (h day^−^^1^); and PEF is the particle emission factor (m^3^ kg^−^^1^).

Cancer risk assessments were calculated by multiplying the dose received with the cancer slope factor (kg day mg^−1^) for oral, dermal or inhalation, respectively. The sum of the pathways is the total cancer risk (Risk_tot_). The parameters used for the calculation of doses are shown in Table 1 [48]. All of the parameters used in the calculation of risk assessment were from the US EPA (2001) [60], and parameters for exposed skin areas assumed the 50th percentile value for the population’s exposure to face, forearms and hands. Many authors used similar parameters for calculations of risk assessment [1,50,51,52,53,54,61].

Cancer risk levels below 10^−6^ were considered insignificant to human health; values between 10^−6^ and 10^−4^ specified a probable carcinogenic effect of PAHs to humans; and levels above 10^−4^ indicated high cancer risk to human via exposure to PAHs [48].

### 2.6. Ecological Risk Assessment of PAHs

In environmental conditions, water can be contaminated with more or less harmful organic compounds. Human exposure to PAHs from water can be estimated using the methodology of the US EPA [62] for residential exposure via ingestion and dermal contact with chemicals in water. Some authors applied this methodology for river waters [40,41] or waters in lakes and lagoons [42,43] or rainwater [44]. In Croatia, most of the population is connected to the public water supply system. Tap water is drinkable and regularly controlled (https://www.hzjz.hr/en/division-for-environmental-health/ accessed on 28 December 2022). Rainwater is used only in some parts of the country in agriculture for irrigation; therefore, the use of the US EPA methodology for assessing human risk due to water consumption on PAHs in bulk deposition samples would not be appropriate.

The occurrence of toxic PAHs in the aquatic environment can cause potential risks for flora and fauna. In this study, the ecological risk assessment was employed to estimate the risk of ten PAHs to surrounding organisms as well as the ecosystem. Risk quotient (RQ) was used for evaluating the ecosystem risk by toxic PAHs in the bulk deposition, which was calculated as follows:

(4) $$\mathrm{RQ}=C_{\mathrm{PAHs}}/C_{\mathrm{QV}}$$

where C_PAHs_ represented the mass concentration of certain PAHs in the bulk deposition expressed as the ∑BaP_eq_, and C_QV_ represented the quality values for each PAH. The negligible concentrations (NCs) and the maximum permissible concentrations (MPCs) of PAHs in water recommended by Kalf et al. (1997) [63] and Cao et al. (2010) [64] were used for calculating the RQ_NCs_ and RQ_MPCs_ as follows:

(5) $${\mathrm{RQ}}_{\mathrm{NCs}}=C_{\mathrm{PAHs}}/C_{\mathrm{QV}\left(\mathrm{NCs}\right)}$$

(6) $${\mathrm{RQ}}_{\mathrm{MPCs}}=C_{\mathrm{PAHs}}/C_{\mathrm{QV}\left(\mathrm{MPCs}\right)}$$

where C_QV(NCs)_ represented the quality values of the NCs of PAHs in the water, and C_QV(MPCs)_ represented the quality values of the MPCs of PAHs in the water. The RQ_∑PAHs(NCs)_ and were defined as follows [43]:

(7) $${\mathrm{RQ}}_{\sum^{\;}\mathrm{PAHs}\left(\mathrm{NCs}\right)}=\sum^{\;}{\mathrm{RQi}}_{\left(\mathrm{NCs}\right)}\left({\mathrm{RQi}}_{\left(\mathrm{NCs}\right)}\geq 1\right)$$

(8) $${\mathrm{RQ}}_{\sum^{\;}\mathrm{PAHs}\left(\mathrm{MPCs}\right)}=\sum^{\;}{\mathrm{RQi}}_{\left(\mathrm{MPCs}\right)}\left({\mathrm{RQi}}_{\left(\mathrm{MPCs}\right)}\geq 1\right)$$

## 3. Results and Discussion

### 3.1. Carcinogenic Activity of PAHs in Atmospheric Particulate Matter and Bulk Deposition

**Figure 1:**
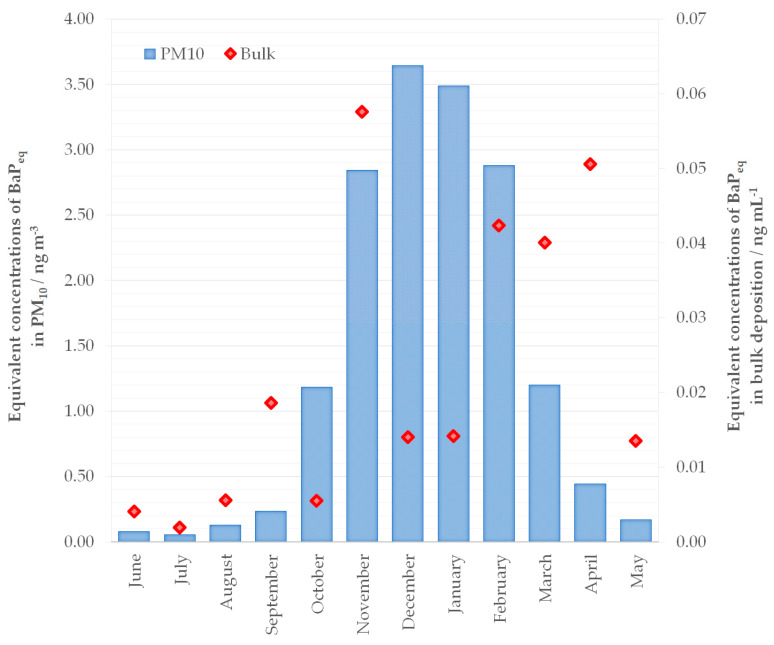
Monthly average equivalent ∑BaP_eq_ mass concentrations of PAHs in the PM_10_ particle fraction and bulk deposition.

To quantify the PAH carcinogenic activity of particulate matter and bulk deposition, the TEFs proposed by Nisbet et LaGoy (1992) [58] were used for calculating the toxic equivalents of individual PAHs relative to BaP (BaP_eq_). The carcinogenic activity of PAHs, expressed as the monthly average equivalent ∑BaP_eq_ mass concentrations of PAHs in the PM_10_ particle fraction and bulk deposition during the measurement period are shown in Figure 1. Average equivalent BaP_eq_ mass concentrations of individual toxic PAHs in the PM_10_ particle fraction and bulk deposition are shown in Table S1 of the Supplementary Material section.

The monthly average of total equivalent BaP_eq_ mass concentrations of PM_10_ varied from 0.057 ng m^−3^ during July to 3.656 ng m^−3^ during December, and the annual ∑BaP_eq_ average was 1.348 ng m^−3^. Concentrations of ∑BaP_eq_ were higher from October to February, while during March to September concentrations decreased, and the lowest values were observed during summer months (Figure 1). The monthly equivalent BaP_eq_ mass concentrations of individual PAHs in the PM_10_ particle fraction are given in Table S1 of the Supplementary Material section. The ∑BaP_eq_ concentrations found in this study were slightly higher than the ones found during cold seasons in a Bangkok metropolitan area [65]. Contrarily, about twice higher ∑BaP_eq_ concentrations were observed in Rome during 1996/1997 [66] and slightly higher in Nagasaki city [67] than the concentrations obtained in this study. Statistical analysis showed differences (*p* < 0.05) of total equivalent ∑BaP_eq_ mass concentrations in the PM_10_ particle fraction between cold months (January, October, November, December) and moderately warm (February, March, April) and warm months (June, July, August, September). Data analysis by seasons showed no differences between autumn and winter or between spring and summer, but differences were found between cold (autumn and winter) and warm periods (spring and summer). During the cold period, mass concentrations of PAHs were higher than in the warm period because of emissions from households; in the study area, residential heating relied mostly on gas, oil or wood, so seasonal differences were expected. Various meteorological parameters such as temperature, precipitation and wind also take part in levels of PAHs in the air. Previous studies showed that air temperature and humidity negatively correlated with PAHs in particle matter while pressure and PAH concentrations positively correlated. However, in bulk deposition air, temperature, humidity and pressure were not correlated with depositions of PAHs while a positive correlation was found with precipitation [18,68,69,70]. The monthly average of air temperature and summarized precipitation during one month are shown in Figure S1 of the Supplementary Material section.

To the best of our knowledge, this paper is the first to present an assessment of the carcinogenic activity of PAHs in a bulk deposition sample using the TEF value, expressed as BaP_eq_. We found only one study that used the TEF approach to assess the risk from PAHs in dry deposition samples [16]. Total equivalent BaP_eq_ mass concentrations of bulk deposition varied from 1.94 ng L^−1^ during July to 57.60 ng L^−1^ during November, and the average ∑BaP_eq_ during the whole measurement period was 22.34 ng L^−1^. The temporal variations in ∑BaP_eq_ in bulk deposition did not follow a regular seasonal cycle as observed in the study by Feng et al. (2017), Garban et al. (2002) and Moon et al. (2006) [20,71,72] where the highest deposition fluxes of the PAHs were observed in winter and the lowest in summer. Mass concentrations of ∑BaP_eq_ in bulk deposition samples were higher during November, February, March and April. In the summer months as well as in October, the total equivalent BaP_eq_ mass concentrations in deposition samples were very low. Due to the small number of samples, data for bulk depositions were tested statistically only between cold (October–March) and warm periods (April–September), and differences were found only for Flu (*p* < 0.05). Levels of Flu were much higher during winter probably due to increased emissions from household heating. The average BaP_eq_ of each toxic PAH taken individually varied from 0.06 ng L^−1^ (Pyr) to 11.45 ng L^−1^ (BaP). The monthly equivalent BaP_eq_ mass concentrations of individual PAHs in the bulk deposition are given in Table S1 of the Supplementary Material section. The larger differences in monthly average equivalent ∑BaP_eq_ mass concentrations of PAHs in bulk deposition samples during the measurement period, as shown in Figure 1, were a consequence of the PAH emission sources and the local meteorological conditions such as temperature, wind speed and precipitation [27].

**Figure 2:**
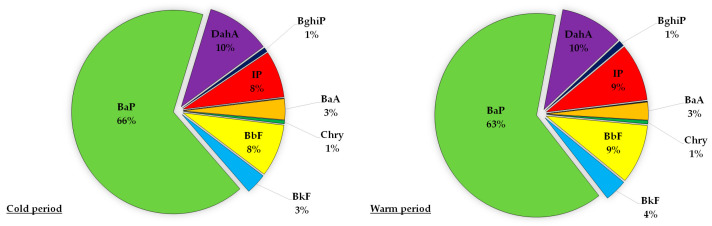
Contribution of individual PAHs to the total carcinogenic activity for PM_10_ samples during the cold and warm periods. Contributions of Flu and Pyr were less than 1%.

**Figure 3:**
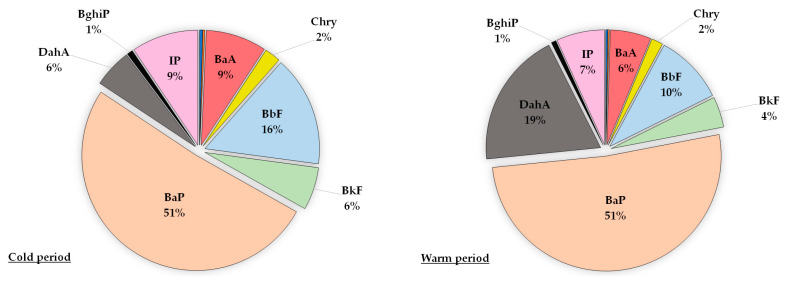
Contribution of individual PAHs to the total carcinogenic activity for bulk deposition samples during the cold and warm periods. Contributions of Flu and Pyr were less than 1%.

Based on the calculated BaP_eq_ levels for specific carcinogenic PAHs, percent contributions of individual PAHs in carcinogenic activity were also calculated and shown in Figure 2 and Figure 3. For PM_10_, the contribution of BaP, the most studied PAH often used as the reference compound, was 63% in warm periods and 66% in cold periods. During both the cold and warm periods, BaP was the highest carcinogenic contributor in PM_10_ followed by DahA, BbF and IP. The lowest contribution, less than 1%, came from Flu and Pyr. A previous study at same location but over a shorter sampling period (one month per each season 2014) for the PM_10_ particle fraction showed a slightly lower contribution of BaP in carcinogenic activity for the overall measured period (54%) [38]. The contribution of BaP to carcinogenic activity in the study by Pehnec and Jakovljević (2018) [38] ranged from 49% to 62% for spring and winter, respectively. The results obtained in this study are in agreement with results from Delgado-Saborit et al. (2011) [73] and Norramit et al. (2005) [65], confirming the importance of BaP as a prominent carcinogenic compound of PAH mixtures in the air.

In bulk deposition samples, BaP contribution was lower than in PM_10_ and amounted to 51% in both periods. In bulk deposition samples, the contribution was different during warm and cold periods. In the cold period, BaP had the highest contribution followed by BbF with 16%, BaA and IP with 9% and BkF and DahA with 6%. In the warm period, BaP was also the highest contributor followed by DahA with 19%, BbF with 10% and IP with 7%. The lowest contribution, less than 1%, came from Flu and Pyr. As this paper presented the first results of the contribution of individual PAHs to carcinogenic activity for a bulk deposition, we could not compare our results with similar studies due to the fact that by now there was no other study assessing the carcinogenic potency of PAHs in the bulk deposition samples expressed as Bap_eq_. A study by He et al. (2017) evaluated the carcinogenic potency of PAHs in the particle dry deposition samples, and results showed that the BaP_eq_∑15-PAHs of dry deposition was highest in the winter where the highest contribution came from BaP and DahA [16].

As shown in Figure 2 and Figure 3, BaP was the most dominant contributor to the total carcinogenic activity for PM_10_ and bulk deposition samples with no significant differences between cold and warm periods, so it can be regarded as a good representative for evaluating the carcinogenicity of the PAH mixture. BaP showed a higher contribution to the total carcinogenic potential in the PM_10_ particle fraction than in the bulk deposition. After the highest percentage contribution of BaP, the next dominant contributor in the PM_10_ was DahA (10%) regardless of whether the period was cold or warm, while in the bulk deposition dominant contributor was DahA (19%) in the warm period and BbF (16%) in the cold period. This deviation in the contribution of individual PAHs in the mixture between PM_10_ and bulk deposition could be a consequence of PAHs variability due to meteorological conditions, especially the amount of precipitation (Figure S1).

### 3.2. Risk Assessment of PAHs

#### 3.2.1. Human Health Risk Assessment

PAHs in the particulate matter were dominantly present in smaller fractions [74,75,76]. A previous study in Zagreb showed that more than 80% of PAHs measured in winter time were bound to the PM_2_._5_ particle fraction while in summer this amounted to more than 60%. Additionally, it was found that more than 90% of PAHs are bound to the PM_1_ particle fraction. Although smaller fractions can reach deeper into the lower respiratory tract and lungs, coarse fractions should not be excluded [77,78]. Eventually, the use of PAHs in PM_10_ instead of in smaller (fine or ultrafine) fractions could lead to some overestimations of the risk.

A human risk assessment using PAH concentration data in PM_10_ was assessed considering inhalation, dermal absorption and ingestion for two different types of residents, for children under the age of 6 and for adults from 20 to 40 at a two-level concentration. The first level was calculated with median concentrations (moderate scenario) of carcinogenic PAHs (∑BaP_eq_), while the second level was based on the maximum concentration (worst scenario). The doses received by these three pathways for PAHs in the PM_10_ particle fraction are shown in Table 2. In the moderate scenario, the highest dose received was through ingestion and was 67.110 ng kg^−1^ day^−1^ for children and 55.925 ng kg^−1^ day^−1^ for adults. The lowest dose received was through inhalation: 0.003 ng kg^−1^ day^−1^ and 0.011 ng kg^−1^ day^−1^ for children and adults, respectively. During the warm period of the year, the dose received was ten times lower than in the cold period for both groups, children and adults. Additionally, in the warm period, the doses received for children were higher than for adults. The lowest dose was for inhalation and the highest was for ingestion. In the worst scenario, the dose received during the cold period was twice higher than in the warm period. The dermal absorption dose was 79.405 ng kg^−1^ day^−1^ and 68.801 ng kg^−1^ day^−1^ for children and adults, respectively. In the warm period, the dermal absorption dose was lower and was 34.949 ng kg^−1^ day^−1^ for children and 30.282 ng kg^−1^ day^−1^ for adults.

Cancer risk is considered as the possibility of developing cancer over a lifetime due to exposure to a probable carcinogen. Cancer risks were calculated multiplying doses with their corresponding carcinogenic slope factor (CSF). Total cancer risk (Risk_tot_) was the sum of the three pathways. The total cancer risks (Risk_tot_) for exposure routes of inhalation, ingestion and dermal absorption from PM_10_ are given in Table 3. According to the literature, cancer risk values of 10^−6^ or less are considered insignificant to human health; values between 10^−6^ and 10^−4^ point to probable carcinogenic effects of PAHs to humans, while levels higher than 10^−4^ imply a high cancer risk to humans [48,60,79]. For PM_10_ exposure during the warm and cold periods, the results of the moderate scenario showed that the lowest cancer risk was obtained for the inhalation pathway, and it was several magnitudes lower than the accepted level (10^−6^), for both children and adults. Risks via ingestion and dermal absorption were higher and exceeded levels of 10^−4^ during the cold period, while during the warm period levels were slightly lower but between 10^−6^ and 10^−4^, which indicated a probable carcinogenic effect to human. The cancer risk levels for the inhalation pathway obtained in this study were several magnitudes higher than the results found in Franco et al. (2017) (risk 10^−13^) [55], who obtained a risk assessment through three pathways and found that cancer risk via inhalation was 104 times less severe than through dermal absorption and ingestion. The results obtained in this paper for the inhalation were much lower than for other two exposure pathways and can be considered negligible. The same results were obtained in two Chinese cities (inhalation risk for children was 1.10 × 10^−6^ and for adults was 2.61 × 10^−9^) [80], but in soil samples collected in Beijing, China results for risk were slightly lower (dermal risk for children was 3.38 × 10^−7^ and for adults 1.37 × 10^−6^) [81].

#### 3.2.2. Ecological Risk Assessment

Atmospheric deposition is one of the main mechanisms of transport of PAHs to all ecosystems, including aquatic ones. The occurrence of PAHs in aquatic environments can cause risks to the aquatic ecosystem. Furthermore, Wright et al. (2018) reported that the deposition of PAHs causes significant damage to plant organisms and is carcinogenic and mutagenic to humans and animals [82]. Mainly, research is focused only on monitoring the deposition process without monitoring its effects on the ecosystem, which means there is a large gap in the understanding of the source and fate of pollutants in the environment.

Potential ecological risk was assesses through a classification of individual PAHs as well as ∑PAHs suggested by Cao et al. (2010) [64]. Furthermore, Cao et al. (2010) suggested that the values of NCs and MPCs of PAHs could be used in other media such as deposition samples, so this risk assessment method was applied in bulk deposition samples [64]. In principle, RQ_NCs_ ≥ 1 and RQ_(MPCs)_ < 1 indicate that the contamination of individual PAHs presents a moderate risk, while RQ_(MPCs)_ ≥ 1 suggests a high ecological risk, (Table 4) and some control actions should be taken. As the distribution of PAH data did not follow a normal distribution, both the mean and median values of RQ_(NCs)_ and RQ_(MPCs)_ were calculated for individual toxic PAHs and ∑PAHs in bulk deposition samples collected in Zagreb, Croatia, in order to avoid possible misinterpretations due to extreme PAH values. However, no significant differences were found between mean and median values, so only the median values of RQ_(NCs)_ and RQ_(MPCs)_ are shown in Table 5. Median values are also better for comparison with other studies, because data distribution of PAHs in environmental samples often deviates from normal.

The median values of RQ_(NCs)_ of each PAHs were higher than the recommended NC values for PAHs in water, except for Flu, Pyr and Chry. RQ_(MPCs)_ of individual PAHs were all less than 1.0. As shown in Table 5, for BaA, BbF and BaP the median values of RQ_(NCs)_ were all more than 1, and RQ_(MPCs)_ values were all less than 1 indicating moderate risk to which the flora and fauna was exposed by being in contact with water containing concentrations of the PAHs collected in the bulk deposition samples. There were no values for RQ_∑PAHs(MPCs)_ due to the fact that RQ_(MPCs)_ > 1 were summed to calculate risk quotients for the sum of toxic PAHs. Overall, there is a moderate ecological risk from some PAHs in bulk deposition which stresses the need for further investigation at other locations as well as during long periods.

## 4. Conclusions

The carcinogenic activity of PAHs in the PM_10_ particle fraction and bulk deposition in Zagreb, Croatia, was assessed based on toxic equivalence factors and equivalent BaP concentrations. Statistically significant differences (*p* < 0.05) in total equivalent ∑BaP_eq_ mass concentrations in PM_10_ particle fraction were found between cold and warm months, while for bulk deposition statistically significant differences were found only for Flu. The most studied PAH in the PM_10_ particle fraction, BaP, contributed to the total PAH carcinogenic activity with 63% in the warm and 66% in the cold period while in bulk deposition samples its contribution was lower (51% for both periods). The human health risk via PAH compounds was evaluated via ingestion, dermal absorption and inhalation using PM_10_-bound PAHs and the literature data. Dermal absorption implied the greatest potential cancer risk, followed by ingestion and inhalation. Cancer risk was slightly higher for children than for adults. Results of this study showed that the population of the investigated area could possibly develop cancer over a lifetime due to exposure to PAHs. The potential ecological risk of PAHs in bulk deposition was estimated based on the risk quotient approach for the first time. A moderate ecological risk for BaA, BbF and BaP was observed, which pointed out the need for further studies.

## Figures and Tables

**Table 1:** Parameters used to calculate cancer risk.

Variable Definition	Symbol	Units	Children	Adults
Body weight	BW	kg	20	80
Exposure frequency	EF	day year^−1^	350	350
Exposure duration	ED	year	6	40
Ingestion rate	IRs	mg day^−1^	200	100
Dermal exposure area	SA	cm^2^	3107	4615
Dermal adherence factor	AF	mg cm^−2^	0.20	0.07
Dermal absorption factor	ABS	unitless	0.13	0.13
Averaging life span	AT	day	25550	25550
Inhalation rate	IRα	m^3^ h^−1^	1.3	3.3
Exposure time	ET	h day^−1^	8	8
Particle emission factor	PEF	m^3^ kg^−1^	1.36 × 10^9^	1.36 × 10^9^
Ingestion carcinogenic slope factor	CSFing	(mg kg^−1^ day^−1^)^−1^	7.3	7.3
Dermal carcinogenic slope factor	CSFderm	(mg kg^−1^ day^−1^)^−1^	25	25
Inhalation carcinogenic slope factor	CSFinh	(mg kg^−1^ day^−1^)^−1^	3.14	3.14

**Table 2:** The dose received (ng kg^−1^ day^−1^) through ingestion, dermal absorption and inhalation for carcinogenic PAHs in the PM_10_ particle fraction.

		Children		Adults	
		Central	Worst	Central	Worst
Cold period	D_ing_	67.110	196.592	55.925	163.827
	D_der_	27.107	79.405	23.487	68.801
	D_inh_	0.003	0.008	0.011	0.032
Warm period	D_ing_	6.798	86.528	5.665	72.106
	D_der_	2.746	34.949	2.379	30.282
	D_inh_	0.0003	0.003	0.001	0.014

**Table 3:** Cancer risk for exposure routes of inhalation, ingestion and dermal absorption from PAHs in PM_10_.

		Children		Adults	
		Central	Worst	Central	Worst
Cold period	Risk_ing_	4.90 × 10^−4^	1.44 × 10^−3^	4.08 × 10^−4^	1.20 × 10^−3^
	Risk_der_	6.78 × 10^−4^	1.99 × 10^−3^	5.87 × 10^−4^	1.72 × 10^−3^
	Risk_inh_	8.06 × 10^−9^	2.36 × 10^−8^	3.41 × 10^−8^	9.99 × 10^−8^
	Risk_tot_	1.17 × 10^−3^	3.42 × 10^−3^	9.95 × 10^−4^	2.92 × 10^−3^
Warm period	Risk_ing_	4.96 × 10^−5^	6.32 × 10^−4^	4.14 × 10^−5^	5.26 × 10^−4^
	Risk_der_	6.86 × 10^−5^	8.74 × 10^−4^	5.95 × 10^−5^	7.57 × 10^−4^
	Risk_inh_	8.16 × 10^−4^	1.04 × 10^−8^	3.45 × 10^−9^	4.40 × 10^−8^
	Risk_tot_	1.18 × 10^−4^	1.51 × 10^−3^	1.01 × 10^−4^	1.28 × 10^−3^

**Table 4:** Risk classification of individual PAHs and ∑PAHs [64]. NCs and MPCs represented the negligible concentrations and the maximum permissible concentrations of PAHs in water recommended by Kalf et al. (1997) [63] and Cao et al. (2010) [64].

Individual PAHs			∑PAHs		
	RQ_NCs_	RQ_MPCs_		RQ_∑PAHs(NCs)_	RQ_∑PAHs(MPCs)_
Risk-free	0		Risk-free	0	
			Low-risk	≥1; <800	0
Moderate-risk	≥1	<1	Moderate-risk_1_	≥800	0
			Moderate-risk_2_	<800	≥1
High-risk		≥1	High-risk	≥800	≥1

**Table 5:** Calculated median values of RQ_(NCs)_ and RQ_(MPCs)_ for individual toxic PAHs and ∑PAHs in bulk deposition.

PAHs	RQ_(NCs)_	RQ_(MPCs)_
Flu	0.0	0.0
Pyr	0.1	0.0
BaA	13.5	0.1
Chry	0.1	0.0
BbF	21.8	0.2
BkF	2.2	0.0
BaP	14.4	0.1
DahA	0.3	0.0
BghiP	0.4	0.0
IP	3.4	0.0
∑PAHs	55.3	-

## Data Availability

Not applicable.

## Supplementary Materials

The following supporting information can be downloaded at: https://www.mdpi.com/article/10.3390/toxics11030228/s1, Figure S1: Meteorological conditions in Zagreb, Croatia during the sampling period (June 2020–May 2021); Table S1: Average equivalent BaP_eq_ mass concentrations of individual toxic PAHs in: (a) the PM_10_ particle fraction and (b) bulk deposition.
