# Supplementary material for: Carcinogenic Activity and Risk Assessment of PAHs in Ambient Air: PM10 Particle Fraction and Bulk Deposition
Source: Toxics. 2023 Feb 27;11(3):228. doi: 10.3390/toxics11030228 (PMC10051387; doi:10.3390/toxics11030228)
Supplement: Supplementary file 1 [file toxics-11-00228-s001.zip › toxics-2163559-supplementary.pdf]

Supplementary Material for "Carcinogenic activity and risk assessment of PAHs in ambient air: PM_{10} particle fraction and bulk deposition"

Toxics

Ivana Jakovljević [1], Iva Smoljo [1,*], Zdravka Sever Štrukil [1] and Gordana Pehnec [1]

[1]Environmental Hygiene Unit, Institute for Medical Research and Occupational Health, 10000 Zagreb, Croatia

[*]Correspondence:

Iva Smoljo

Institute for Medical Research and Occupational Health

Ksaverska c. 2, 10000 Zagreb, Croatia

E-mail: ismoljo@imi.hr

Table S1. Average equivalent BaP_{eq} mass concentrations of individual toxic PAHs in: a) the PM_{10} particle fraction and b) bulk deposition

**a)**	ng m^{-3}										
	Flu	Pyr	BaA	Chry	BbF	BkF	BaP	DahA	BghiP	IP	∑BaPeq
June	0.00004	0.00004	0.003	0.001	0.008	0.003	0.048	0.009	0.001	0.007	0.079
July	0.00002	0.00002	0.002	0.0004	0.006	0.002	0.034	0.007	0.001	0.006	0.057
August	0.0001	0.0001	0.004	0.001	0.012	0.005	0.076	0.020	0.001	0.011	0.130
September	0.0001	0.0001	0.006	0.001	0.021	0.008	0.156	0.021	0.002	0.021	0.236
October	0.0003	0.0003	0.025	0.004	0.097	0.038	0.804	0.102	0.009	0.103	1.183
November	0.001	0.001	0.089	0.015	0.230	0.091	1.912	0.276	0.018	0.211	2.843
December	0.001	0.001	0.119	0.021	0.307	0.121	2.418	0.372	0.024	0.265	3.647
January	0.001	0.001	0.128	0.021	0.295	0.117	2.319	0.320	0.024	0.261	3.488
February	0.001	0.001	0.108	0.016	0.243	0.097	1.873	0.328	0.020	0.196	2.882
March	0.0002	0.0002	0.034	0.005	0.103	0.043	0.748	0.161	0.010	0.096	1.200
April	0.0002	0.0002	0.012	0.002	0.043	0.016	0.290	0.035	0.004	0.040	0.443
May	0.0001	0.0001	0.005	0.001	0.015	0.006	0.103	0.021	0.001	0.016	0.168
**b)**	ng mL^{-1}										
	Flu	Pyr	BaA	Chry	BbF	BkF	BaP	DahA	BghiP	IP	∑BaPeq
June	0.021	0.029	0.271	0.099	0.562	0.333	2.367	0.000	0.033	0.381	4.096
July	0.069	0.029	0.119	0.097	0.368	0.204	0.836	0.000	0.017	0.206	1.945
August	0.040	0.018	0.621	0.111	0.655	0.000	3.601	0.000	0.052	0.480	5.578
September	0.034	0.030	1.478	0.229	1.924	0.886	10.693	1.999	0.108	1.253	18.633
October	0.031	0.015	0.325	0.128	0.833	0.401	2.802	0.338	0.055	0.566	5.494

November	0.166	0.123	5.449	1.439	10.730	3.970	28.479	0.000	0.549	6.696	57.602
December	0.185	0.095	1.965	0.584	2.759	1.026	4.929	0.924	0.101	1.433	14.001
January	0.123	0.075	1.221	0.453	2.440	0.863	7.405	0.000	0.118	1.486	14.186
February	0.175	0.111	3.711	0.791	6.138	2.566	24.845	0.000	0.369	3.680	42.387
March	0.077	0.067	2.199	0.529	4.179	1.615	20.440	8.196	0.246	2.501	40.047
April	0.080	0.069	2.381	0.835	4.444	1.896	24.045	13.438	0.289	3.099	50.575
May	0.030	0.016	0.557	0.152	1.321	0.743	6.994	2.598	0.139	0.993	13.543

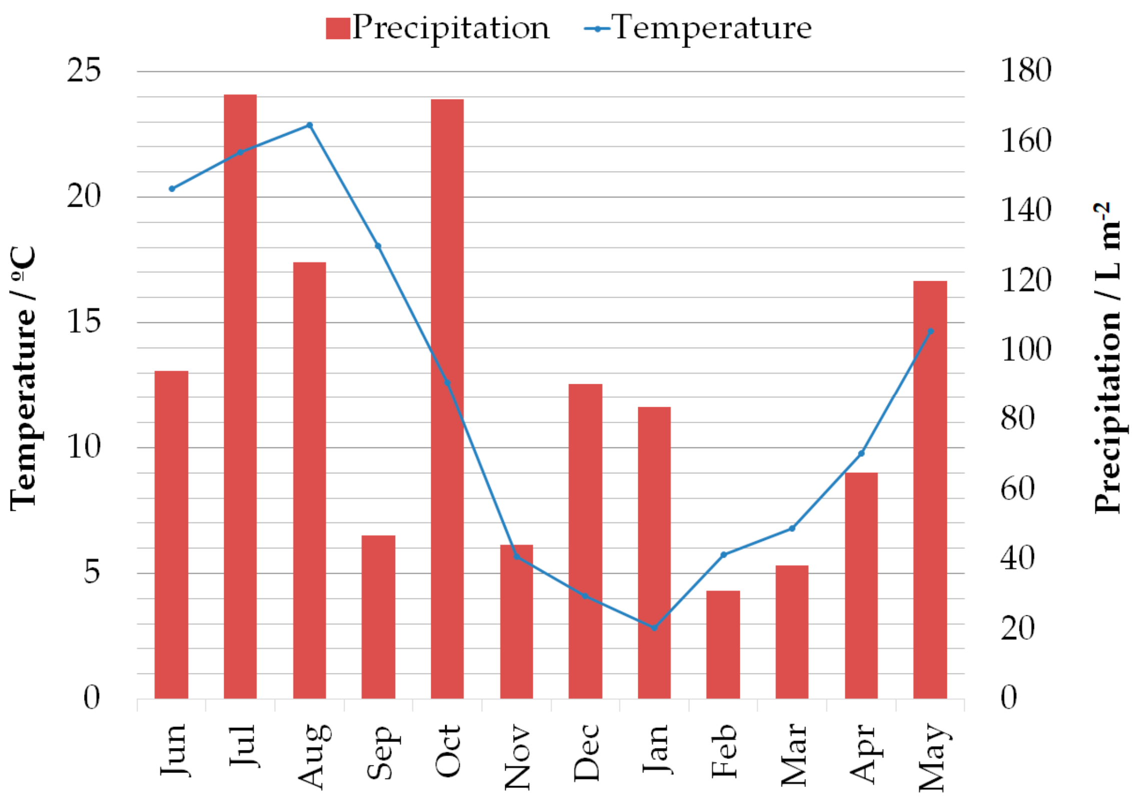

Figure S1. Meteorological conditions in Zagreb, Croatia during the sampling period (June 2020 - May 2021).
